# Insulin Treatment Is Associated With Improved Fetal Placental Vascular Circulation in Obese and Non-obese Women With Gestational Diabetes Mellitus

**DOI:** 10.3389/fendo.2019.00084

**Published:** 2019-02-27

**Authors:** Giulia Barda, Jacob Bar, Margarita Mashavi, Letizia Schreiber, Marina Shargorodsky

**Affiliations:** ^1^Department of Obstetrics and Gynecology, Edith Wolfson Medical Center, Holon, Israel; ^2^Sackler Faculty of Medicine, Tel Aviv University, Tel Aviv, Israel; ^3^Department of Medicine, Edith Wolfson Medical Center, Holon, Israel; ^4^Department of Pathology, Edith Wolfson Medical Center, Holon, Israel; ^5^Department of Endocrinology, Edith Wolfson Medical Center, Holon, Israel

**Keywords:** insuli, diabetes gestational, obesity, gestational hypertension, placental circulation

## Abstract

**Objective:** The present study was designed to investigate the impact of carbohydrate restriction and insulin treatment on placental maternal and fetal vascular circulation in obese and non-obese women with gestational diabetes mellitus (GDM).

**Design and methods:** One Hundred Ninety-One women with GDM who gave birth and underwent a placental histopathological examination at Wolfson Medical Center, Israel, were included in the study: 122 women who were treated with carbohydrate/calorie restriction diet (Group 1) and 69 women who were treated with diet plus insulin (Group 2). Additionally, each group was divided into two subgroups according to pre-pregnancy BMI: non-obese and obese.

**Results:** Maternal vascular malperfusion lesions did not differ significantly between groups. Vascular lesions related to fetal malperfusion were significantly lower in GDM women treated by insulin and diet compared to women with diet alone (*p* = 0.027). Among fetal malperfusion lesions, villous changes consistent with fetal thrombo-occlusive disease (FTOD) were significantly lower in women treated with diet plus insulin and lowest in GDM women with pre-pregnancy BMI < 30 kg/m^2^ (*p* = 0.009). In the logistic regression analysis, insulin treatment was significantly associated with a decreased rate of villous changes consistent with FTOD (OR 0.97, 95% CI 0.12–0.80, *p* = 0.03). Prevalence of gestational hypertension was higher in obese women of both treatment groups (*p* = 0.024).

**Conclusion:** Combination of obesity and GDM increased rate of FTOD and prevalence of gestational hypertension. Carbohydrate restriction diet plus insulin treatment was associated with improved fetal placental vascular circulation, especially in GDM women with pre-pregnancy BMI < 30 kg/m^2^.

## Introduction

Gestational diabetes mellitus (GDM) is a disease associated with maternal glucose intolerance, fetal hyperglycaemia/hyperinsulinaemia, abnormal placental vascular function, and adverse perinatal outcomes (1–3). Treatment of GDM with diet management to control for maternal glycaemia usually reduces glucose levels to the recommended levels (4). Although diet management in GDM pregnancies results in both the mother and the newborn being normoglycaemic at birth, several alterations in terms of fetoplacental vascular reactivity are evident (5, 6). Moreover, recent studies have found abnormal endothelial function due to altered expression of insulin receptors (IR) in the fetoplacental endothelium of pregnant women who underwent diet management, even with adequately controlled GDM (7). These adverse changes in placental structure and function have harmful consequences for neonatal and maternal pregnancy outcome.

A percentage of GDM women do not achieve the recommended values of glycaemia with diet management, and consequently receive insulin therapy until delivery. It has been shown that insulin treatment for GDM restores placental insulin receptors expression, leading to normalization of endothelial function. Moreover, placental insulin resistance, associated with a reduction in phosphorylated AKT (p-AKT), found in diet treated GDM, can be reversed by insulin treatment during pregnancy (8, 9).

Thus, therapeutic interventions in GDM pregnancies are crucial not only for the control of maternal and fetal glycaemia, but also for the prevention of fetoplacental vascular dysfunction as well as the subsequent risk reduction of neonatal complications and adulthood metabolic diseases (e.g., obesity, hyperlipidemia, and type 2 diabetes mellitus). Nevertheless, the impact of GDM treatment on placental histopathology has not been examined. The present study was designed to investigate the impact of insulin treatment on maternal and fetal placental vascular circulation, pregnancy complications, and neonatal outcome in obese, and non-obese women with GDM.

## Methods

One Hundred Ninety-One women with GDM who gave birth and underwent a placental histopathological examination between 2007 and 2013 at Wolfson Medical Center were included in the study. Exclusion criteria included pre-gestational type 1 and type 2 diabetes mellitus, multiple pregnancies, abnormal fetus karyotype, and labor before 37 weeks gestation.

Early pregnancy screening for gestational diabetes by the patient's medical history and fasting glucose levels was performed. If the result of initial testing was negative, a repeat screening at 24–28 weeks of gestation was performed.

Following the diagnosis of GDM, diet management was initiated. Diet management included a carbohydrate restricted diet (~200 g carbohydrates per day maximum), which was focused on optimizing participants' consumption of vegetables, fruits, whole-grain products, low-fat dairy products, and a lower intake of sugar-rich foods. If treatment of GDM with diet management did not sufficiently reduce glucose levels to the recommended levels (fasting blood glucose <105 mg/dl) during the first trimester, insulin treatment (short- or long-lasting insulin) was initiated.The study consisted of two groups according to type of intervention: Group 1 included 122 pregnant women with GDM who were treated with diet management, while group 2 included 69 pregnant women with GDM who received diet management together with insulin treatment. Additionally, each group was divided into two subgroups according to pre-pregnancy BMI: Subgroup A included non-obese GDM women (BMI < 30 kg/m^2^) and subgroup B contained obese pregnant women (BMI ≥30 kg/m^2^).

The present study was approved by the ethics committee of the Edith Wolfson Institutional Review Board, Wolfson Medical Center, Israel. Since the study was observational, retrospective cohort study, informed consent from the participants of this study was not required.

### Placental Examination

**Figure 1:**
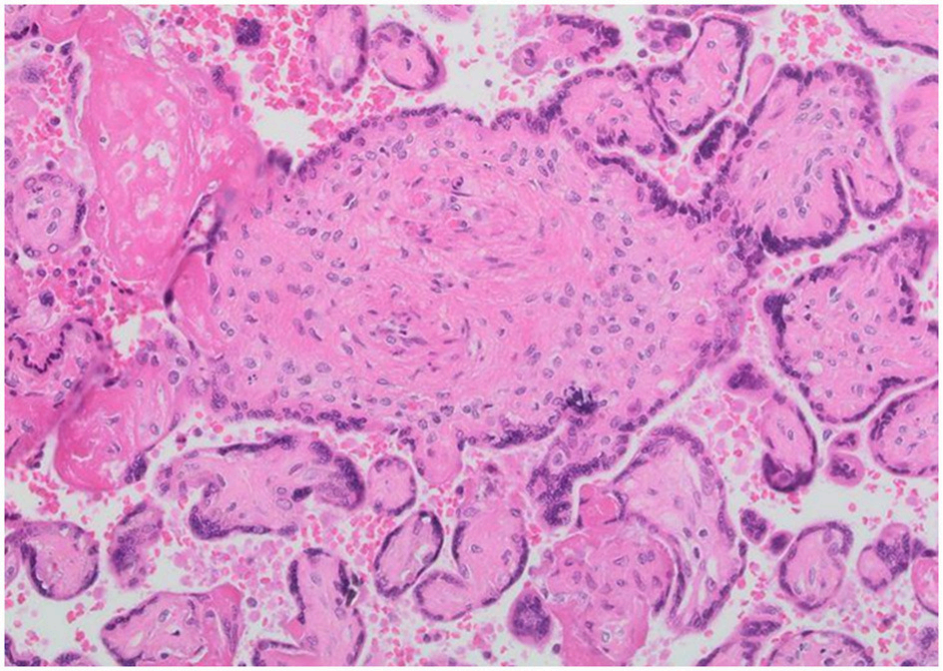
Representative images of fetal placental vascular circulation abnormalities. Villous changes consistent with fetal thrombo-occlusive disease.

Placental histology was analyzed according to the criteria of the Society of Pediatric Pathology (10) with the 2016 Amsterdam Placental Workshop modifications (11). Placental findings were divided into maternal and fetal malperfusion lesions. Maternal mulperfusion lesions included marginal and retro-placental hemorrhages, vascular (i.e., acute atherosis and mural hypertrophy) and villous changes (i.e., villous infarcts, increased syncytial knots, and intervillous fibrin deposition). Fetal malperfusion lesions included vascular and villous lesions related to thrombo-occlusive disease. A representative image of fetal malperfusion abnormalities is shown in Figure 1. A single pathologist, who was blind to the type of gestational diabetes treatment and BMI, performed all the placental pathological examinations, using a previously described standard protocol (12, 13).

### Statistical Analysis

Analysis of data was carried out using SPSS 9.0 statistical analysis software (SPSS Inc., Chicago, IL, USA, 1999). The Kolmogorov-Smirnov test was used for normalcy of distribution of continuous variables (cut off at *p* = 0.01). Categorical variables such as sex and co-morbidities were described using frequency distributions and are presented as frequency (%). Differences across the study groups were examined by one-way analysis of variance (ANOVA). Variables for which across-group differences were detected underwent *post hoc* pairwise testing using the Bonferroni test. The General linear model (GLM) was developed using a backward, stepwise approach and inclusion of variables is this model was based on univariate results.

## Results

Demographic and clinical characteristics of the four study groups according to type of treatment intervention and pre-pregnancy BMI are presented in Table 1. As shown, four groups were similar in terms of age (*p* = 0.938), gravidity (*p* = 0.661), and parity (*p* = 0.199). Pre-gestational BMI did not differ significantly between groups 1A and 2A (*p* = 0.311) as well as 1B and 2B (*p* = 0.981). Weight gain during pregnancy did not differ significantly between the study groups (*p* = 0.062). Mean fasting plasma glucose during the first trimester of pregnancy differed significantly between the study groups, and was significantly higher in insulin plus diet treated obese women (*p* = 0.040). Mean fasting plasma glucose did not differ significantly between groups during the second and third trimester of pregnancy.

**Table 1:** Maternal and neonatal characteristics according to the study groups.

**Variables**	**Group 1**		**Group 2**		***p*-value**
	**Insulin treatment**		**Diet management**		
	**Non-obese (A)**	**Obese (B)**	**Non-obese (A)**	**Obese (B)**	
Age (y)	32.5 ± 4.7	32.5 ± 4.6	32.0 ± 5.3	32.0 ± 5.5	0.938
Gravidity	2.9 ± 1.9	2.9 ± 1.6	2.6 ± 1.7	3.0 ± 2.1	0.661
Parity	1.1 ± 1.4	1.3 ± 1.2	0.9 ± 1.0	1.2 ± 1.2	0.199
Pregestational BMI (kg/m^2^)	25.3 ± 3.1	36.7 ± 5.3	23.8 ± 3.0	35.4 ± 4.1	0.001
Weight at delivery (kg)	76.9 ± 17.3	90.5 ± 47.2	54.9 ± 33.6	73.0 ± 53.9	0.001
Weight gain during pregnancy (kg)	12.8 ± 6.4	10.3 ± 8.0	12.1 ± 6.7	8.7 ± 7.8	0.062
Gestational hypertension, *n* (%)	2 (6.1)	5 (13.9)	1 (1.3)	6 (13.9)	0.024
Preeclampsia, *n* (%)	2 (6.1)	1 (2.8)	6 (7.6)	8 (18.6)	0.067
Smoking, *n* (%)	5 (15.6)	6 (16.7)	10 (12.8)	8 (18.6)	0.853
Family history of diabetes, *n* (%)	15 (45.5)	17 (47.2)	33 (41.8)	16 (37.2)	0.812
Family history of hypertension, *n* (%)	5 (15.2)	9 (25.0)	15 (19.0)	5 (11.6)	0.452
1st trimester fasting glucose (mg/dl)	95.9 ± 16.2	103.6 ± 23.6	91.6 ± 18.8	97.0 ± 17.5	0.040
2nd trimester fasting glucose (mg/dl)	95.5 ± 22.5	115.5 ± 31.6	101.6 ± 27.3	104.2 ± 27.3	0.050
3rd trimester fasting glucose (mg/dl)	96.7 ± 16.4	101.6 ± 22.1	93.6 ± 28.1	100.0 ± 27.6	0.711
Birth weight (g)	3302.2	3568.4	3260.7	3570.5	0.823
Umbilical cord PH	7.0 ± 1.4	7.3 ± 0.1	7.3 ± 0.1	7.1 ± 1.2	0.435
Apgar at 1 min	8.8 ± 0.1	8.7 ± 0.2	8.6 ± 0.2	8.6 ± 0.2	0.796
Apgar at 5 min	9.9 ± 0.4	9.8 ± 0.2	9.9 ± 0.1	9.9 ± 0.2	0.456
Large for gestational age, *n* (%)	5 (15.1)	13 (36.1)	20 (25.3)	17 (39.5)	0.078
Macrosomia, *n* (%)	2 (6.1)	7 (19.4)	11 (13.9)	10 (23.3)	0.192
Hypoglycemia, *n* (%)	4 (12.1)	6 (17.1)	10 (12.7)	2 (4.7)	0.366
Neonatal Intensive Care Unit, *n* (%)	8 (24.2)	14 (40.0)	34 (43.0)	12 (27.9)	0.161

*BMI, body mass index. By-group comparisons made using one way analysis of variance (ANOVA) simultaneously comparing all study groups*.

As can be seen in Table 1, neonatal outcome parameters (i.e., birth weight, large for gestational age (LGA), macrosomia, first and fifth minute Apgar score, hypoglycemia, cord artery pH and ICU admission) did not differ significantly between groups. Among pregnancy complications, prevalence of gestational hypertension was higher in obese women in both treatment groups, compared to GDM women with pre-pregnancy BMI < 30 kg/m^2^ (*p* = 0.024).

### Placental Findings

Placental findings of the study groups according to type of intervention and pre-pregnancy BMI are presented in Table 2. As can be seen, maternal vascular malperfusion (MVM) lesions of the placental bed did not differ significantly between groups (*p* = 0.230), whereas vascular lesions related to fetal malperfusion were significantly lower in insulin plus diet treated patients, obese, and non-obese (Group 1A and 1B) than in diet managed women (Groups 2A and 2B) (*p* = 0.027). Among placental lesions related to fetal vascular malperfusion, villous changes consistent with fetal thrombo-occlusive disease (FTOD) were significantly lower in women treated with diet plus insulin and lowest in GDM women with pre-pregnancy BMI < 30 kg/m^2^ (*p* = 0.009). Multiple regression analysis was arrived at using a backward, stepwise approach to identify variables independently associated with composite fetal malperfusion abnormalities. Variables were included in the model based on their associations in univariate analyses: mean fasting plasma glucose during the first trimester of pregnancy, gestational hypertension and insulin treatment. In this model, insulin plus diet treatment was significantly associated with a decreased rate of villous changes consistent with composite FVM (OR 0.35, 95% CI 0.12–0.99, *p* = 0.049). In the regression model of villous changes consistent with fetal thrombo-occlusive disease were included variables based on their associations in univariate analyses (mean fasting plasma glucose during the first trimester of pregnancy and insulin treatment). In this model, diet management with insulin treatment was significantly associated with a decreased rate of villous changes consistent with FTOD (OR 0.97, 95% CI 0.12–0.80, *p* = 0.030).

**Table 2:** Histopathological characteristic of placenta according to the study groups.

**Variables**	**Group 1**		**Group 2**		
	**Insulin treatment**		**Diet management**		
	**Non-obese (A)**	**Obese (B)**	**Non-obese (A)**	**Obese (B)**	
Placental weight (g)	503 ± 138.4	599 ± 136.0	521 ± 120.2	571 ± 125.9	0.003
Fetal Placental Ratio	8.3 ± 10.8	6.1 ± 0.9	6.5 ± 1.2	6.4 ± 0.8	0.158
Composite MVM rate, *n* (%)	19 (57.5)	13 (36.1)	42 (53.2)	19 (44.2)	0.230
Vascular lesions related to maternal malperfusion	2 (6.1)	2 (5.6)	7 (8.9)	2 (4.6)	0.811
Villous lesions related to maternal malperfusion	17 (51.5)	11 (30.6)	37 (46.8)	17 (39.5)	0.268
Composite FVM rate, *n* (%)	2 (6.1)	3 (8.3)	11 (13.9)	12 (27.9)	0.027
Vascular lesions with FTOD, *n* (%)	2 (6.1)	2 (5.6)	2 (2.5)	4 (9.3)	0.449
Villous lesions with FDOD, *n* (%)	0 (0.0)	1 (2.8)	9 (11.4)	9 (20.9)	0.009
MIR *n* (%)	4 (12.1)	1 (2.8)	9 (11.4)	5 (13.9)	0.467
FIR *n* (%)	3 (9.1)	0 (0.0)	5 (6.3)	2 (4.7)	0.590

*By-group comparisons made using one way analysis of variance (ANOVA) simultaneously comparing all study groups*.

## Discussion

The major finding of the present study is that carbohydrate restricted diet management with insulin treatment was associated with improved placental vascular circulation of fetal origin in obese and non-obese women with GDM. Additionally, villous changes consistent with fetal thrombo-occlusive disease (FTOD) were lowest in GDM women with pre-pregnancy BMI < 30 kg/m^2^ treated with diet plus insulin. Combination of obesity and GDM increased rate of villous changes consistent with FTOD and prevalence of gestational hypertension in both treatment groups. Thus, prevention of obesity throughout women's reproductive age may translate to improved placental circulation and potential positive effects on adulthood metabolic diseases.

Although insulin is not essential for the placental transfer of glucose, maternal insulin can bind to insulin receptors in the trophoblast membranes, and activate adenosine and insulin receptors (cAMP, PKA, MAPK, and PI3K/Akt) as well as increase placental expression of GLUT-4 and GLUT-9, subsequently affecting placental and fetal development (14, 15). Insulin treatment in GDM could result in restoration of the expression and activity of insulin and adenosine receptors and the l-arginine–NO signaling pathway as well as promote placental fatty acid transfer and overcome placental insulin resistance (7, 16, 17). Whereas, in trophoblast plasma membranes from gestational diabetic women treated with diet alone there is less expression of insulin receptors, in women treated with insulin there is greater expression of insulin receptors (18). The altered expression of IRs in the fetoplacental endothelium of GDM women leads to abnormal fetal microvascular and macrovascular circulation, even when their diet was adequately controlled. Insulin treatment restored IR expression in these patients, leading to normal endothelial function (7, 8). Our results further support previous findings demonstrating that insulin treatment improves insulin resistance, restores placental insulin and adenosine receptors expression, and positively impacts fetoplacental circulation and endothelial function.

It has been shown that women treated with insulin have a higher metabolic risk profile and lower insulin sensitivity, compared to diet-treated women with GDM. Reduced insulin sensitivity and beta-cell function in insulin-treated women, remained significant after adjustment for confounders such as age, BMI, ethnicity, and pregnancy weight gain (19). Impaired beta-cell compensation is probably chronic and leads to alterations in placental structure and function, neonatal complications, and adulthood metabolic diseases (20–22). In the present study, insulin treatment of gestational diabetes, characterized by more severe glucose intolerance, was not associated with an adverse impact on placental vascular circulation. Moreover, a beneficial effect of insulin treatment on fetal placental vascular circulation was observed. Although, mean fasting plasma glucose did not differ significantly between groups during the second and third trimester of pregnancy and in the regression model, mean fasting plasma glucose during the first trimester was not significantly associated with a villous changes consistent with FTOD (OR 0.963, 95% CI 0.919–1.009, *p* = 0.111), the possibility that beneficial effect of insulin therapy in terms of placental vascular circulation is linked to a better diabetes control cannot be excluded. Since women with GDM needing insulin treatment, remain a high risk population, further research is necessary to determine whether beneficial effect on placental vascular circulation could lead to improved neonatal and pregnancy outcomes in this group. In the present study, insulin treatment of gestational diabetes was associated with improvement of placental vascular circulation but not pregnancy complications. However, the fact that both study groups were similar in terms of neonatal complications, could indicate improvement regarding clinical outcomes in insulin treated women because this group is characterized by more severe impairment of glucose homeostasis and we would have expected more pregnancy complications in these patients.

Obesity during pregnancy is associated with impaired endothelial function, increased pro-inflammatory cytokine expression and being associated with a higher risk of placental pathological lesions (23, 24). We previously reported that obesity, *per se*, emerged as a significant independent predictor of fetal vascular malperfusion and Willous maturation defect (25). In the present study, combination of GDM and obesity was associated with increased prevalence of villous changes consistent with fetal thrombo-occlusive disease and gestational hypertension in both treatment groups. Thus, addressing maternal pre-pregnancy BMI and recommending overweight women planning a pregnancy to return to a normal BMI, is reasonable and important.

There are advantages as well as limitations to our study. One advantage is that all placental pathological examinations were performed by a single pathologist, who was unaware of the GDM treatment approach, using validated placental pathological criteria. Another advantage is that the study groups were similar in terms of age, gravidity, parity and mode of delivery, ruling out these factors as confounders. The major limitation of the present study is retrospective cross-sectional design. Thus, prospective long term studies evaluating the impact of different treatment approach for GDM (i.e., diet, insulin, and oral hypoglycemic drugs) are needed to confirm these findings.

In conclusion, our findings favor insulin treatment in terms of placental vascular circulation, and support recently published guidelines indicating insulin as the preferred medication in gestational diabetes treatment. Addressing maternal weight control before and during pregnancy may translate to further improvement of placental vascular circulation and better pregnancy outcomes.

## Data Availability

All datasets generated for this study are included in the manuscript and/or the supplementary files.

## Author Contributions

All authors listed have made a substantial, direct and intellectual contribution to the work, and approved it for publication.

### Conflict of Interest Statement

The authors declare that the research was conducted in the absence of any commercial or financial relationships that could be construed as a potential conflict of interest.
